# What is behind smoker support for new smokefree areas? National survey data

**DOI:** 10.1186/1471-2458-10-498

**Published:** 2010-08-18

**Authors:** Nick Wilson, Deepa Weerasekera, Tony Blakely, Richard Edwards, George Thomson, Heather Gifford

**Affiliations:** 1 Department of Public Health, University of Otago, Wellington, New Zealand; 2 Whakauae Research Services, PO Box 102, Whanganui, New Zealand

## Abstract

**Background:**

Some countries have started to extend indoor smokefree laws to cover cars and various outdoor settings. However, policy-modifiable factors around smoker support for these new laws are not well described.

**Methods:**

The New Zealand (NZ) arm of the International Tobacco Control Policy Evaluation Survey (ITC Project) derives its sample from the NZ Health Survey (a national sample). From this sample we surveyed adult smokers (n = 1376).

**Results:**

For the six settings considered, 59% of smokers supported at least three new completely smokefree areas. Only 2% favoured smoking being *allowed *in all the six new settings. Support among Maori, Pacific and Asian smokers relative to European smokers was elevated in multivariate analyses, but confidence intervals often included 1.0.

Also in the multivariate analyses, "strong support" by smokers for new smokefree area laws was associated with greater knowledge of the second-hand smoke (SHS) hazard, and with behaviours to reduce SHS exposure towards others. Strong support was also associated with reporting having smokefree cars (aOR = 1.68, 95% CI = 1.21 - 2.34); and support for tobacco control regulatory measures by government (aOR = 1.63, 95% CI = 1.32 - 2.01). There was also stronger support by smokers with a form of financial stress (not spending on household essentials).

**Conclusions:**

Smokers from a range of population groups can show majority support for new outdoor and smokefree car laws. Some of these findings are consistent with the use of public health strategies to support new smokefree laws, such as enhancing public knowledge of the second-hand smoke hazard.

## Background

Legislation that bans smoking indoors in public places is now commonplace in developed countries. There is extensive scientific evidence to support these bans being effective in protecting non-smokers from second-hand smoke (SHS) and in contributing to advancing tobacco control in other ways [1-4]. There are a range of other domains for which some jurisdictions have passed smokefree laws, including cars with children; settings where smoke can drift from outside to inside (eg, entranceways and near windows); and various other outdoor settings (eg, outside eating areas of hospitality venues, stadiums, beaches, children's playgrounds and parks).

For some of these new domains the health impacts of SHS remain relevant, especially enclosed settings such as smoking in private cars [5]. But for outdoor bans it has been argued that the central issue is reducing the modelling to children of smoking as a normal behaviour, and thus reduce smoking uptake [6]. Outdoor smokefree policies may also reduce the SHS health hazard [7], reduce nuisance to non-smokers, and reduce fire risk and litter [8].

For jurisdictions with survey data, there appears to be majority public support for laws requiring cars that contain children to be smokefree. In five surveys in 2005 or since (in California, NZ and Australia), this support from *smokers *was 77% or more [9].

In general population surveys reported since the year 2000, *minority *public support has been reported for smokefree parks in the US [10]; for smokefree outdoor bar/club patios in California [11]; for outdoor public spaces in North East England [12]; and for some outdoor public places (for example bus shelters or public parks) in the UK [13]. However, other surveys from 2000 have reported *majority *public support for various outdoor smokefree settings. In California these have included: child play yards, outside building entrances, outdoor restaurant dinning patios, outdoor public places (such as parks, beaches, golf courses, zoos, or sports stadiums) [11,14,15]. Similarly, there has been majority public support for the following being smokefree:

• outdoor areas used by children, during youth events, and in all outdoor parks in Minnesota [16],

• outside building entrances in three Canadian provinces [17],

• children's playgrounds, outside workplace doors/entrances, sports stadiums, beaches, and outdoor dining areas in an Australian state [18,19],

• "near children" in the UK [13].

In some cases there has also been reported majority support among *smokers *eg, for smokefree playgrounds at 83% in Victoria (Australia) [20], and for outdoor areas used by children in Minnesota (at 51%) [16]. But the reasons behind smoker attitudes to such new smokefree areas are not well studied. Understanding these attitudes is potentially important if such understanding can: (i) inform appropriate public health interventions to accelerate the spread of such new smokefree areas; (ii) improve the design of new smokefree laws so that there is adequate public and smoker acceptability and compliance; and (iii) maximise the synergies with other tobacco control interventions (eg, mass media campaigns).

New Zealand is a relatively good place to study such smoker attitudes, as there are no smokefree car laws, and outdoor smokefree areas are still relatively uncommon. The latter are limited to the grounds of schools; the grounds of some hospitals; several stadiums and campuses of tertiary educational institutions; and a minority of parks (ie, around a quarter of local government bodies have smokefree park policies [21]). However, the smokefree park "policies" are only "educative" with no legal status, and one survey found that only 63% of adult users of a "smokefree park" were aware of the local smokefree policy [22]. Nevertheless, the New Zealand situation does involve a relatively comprehensive national *indoor *smokefree law (including all the insides of restaurants, bars and other indoor workplaces). The available evidence strongly indicates majority public support and compliance with this indoor smokefree law [23-25].

In this study we aimed to examine support among smokers for new smokefree areas, and to examine how this support is associated with specific sociodemographic variables, attitudes and behaviours. Our study population of New Zealand smokers allowed us to explore a culturally diverse population with good data on socio-economic position. Specific hypotheses we aimed to test were that support for new smokefree areas would be greater among smokers who: (i) were more knowledgeable about the health hazards of smoking and of the SHS hazard; (ii) reported having smokefree homes and cars; and (iii) supported tobacco control regulatory measures by government. All of these relationships can be potentially modified by governments and organisations interested in advancing tobacco control.

## Methods

### The ITC Project

The International Tobacco Control Policy Evaluation Survey (the ITC Project) is a multi-country study on tobacco use epidemiology and tobacco control policy evaluation. A full description of the ITC Project conceptual framework and methods have been published elsewhere [26,27]. The New Zealand arm of the ITC Project survey differs somewhat from the other ITC Project countries in that the smokers involved are taken from the New Zealand Health Survey (NZHS) participants (with this survey being conducted in 2006/2007). Methods of the NZHS are detailed more fully in the report on the key results [28] and a detailed methods report [29]. Respondents were selected by a complex sample design, which included systematic boosted-sampling of the Māori, Pacific and Asian populations. Interviews were conducted face-to-face in respondents' homes by trained interviewers (on contract to the Ministry of Health) and resulted in a total of 11,924 interviews with respondents aged 18 and over. The overall response rate was 67.9%. Other issues around the NZHS response rate as it relates to the ITC project are detailed in an online *Methods Report *[30].

### Participants

From the NZHS sample we sampled adult smokers who were 18 years or older and who were willing to participate in further research when asked this at the end of the NZHS interview (this was 85.2% of the adult smokers in the NZHS). Out of 2438 potential respondents who met these criteria, a total of 1376 completed the NZ ITC Project Wave 1 questionnaire, giving a response rate of 56.4%. But when considering the NZHS response rate and willingness to further participate, then the overall response rate is reduced further to 32.6% (for details see an online *Methods Report *[30]).

### Procedures

Surveying of these participants was carried out using a computer-assisted telephone survey (sub-contracted to Roy Morgan Research). The first wave of participants was interviewed between March 2007 and February 2008, usually 3-4 months after their NZHS interview. The study protocol was cleared by the Multi-Region Ethics Committee in New Zealand (MEC/06/07/071) and by the Office of Research Ethics, University of Waterloo, Waterloo, Canada (ORE #13547).

### Measures

The six questions on attitudes to new smokefree areas involved asking: "Do you think smoking should be allowed..."; [Cars]: "... in cars with pre-school children in them?"; [Playgrounds]: "... at council-owned playgrounds?"; [Entranceways]: "... within 5 metres of the entrance to public buildings?"; [Beaches]: "... on lifeguard-patrolled beaches?"; [Outdoors at pubs]: "... in some of the outdoor seating areas of pubs?"; [Outdoor eating areas]: "And now thinking about the outdoor eating areas of restaurants and cafés. Do you think that smoking should be allowed in ALL outdoor eating areas, in some outdoor eating areas, or not allowed in outdoor eating areas at all?"

**Figure 1:**
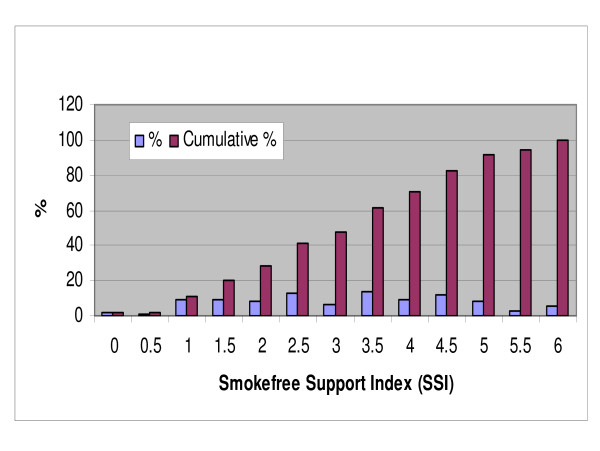
**Distribution of support levels for smokefree areas and outdoor areas by a "smokefree support index" (higher scores mean higher support, weighted results)**.

Using these questions we developed a "smokefree support index" (SSI). Respondents scored: "1.0" if they reported that smoking should not be allowed in a particular setting, "0.5" if they reported that smoking should not be allowed in "some outdoor eating areas" (for the particular question on eating areas); and "0" if they reported that smoking should be allowed or they responded "don't know". The Cronbach's alpha score for this index was 0.65 and the distribution of total scores for the six individual questions is shown in Figure 1.

Some socio-demographic questions were asked in the NZHS but most of the smoking behaviour and smoking-related belief questions were from the questionnaire used in Wave 4 four-country ITC survey. We used some of the indices used elsewhere in ITC Project analyses [31-33], but also developed others for this analysis (see an online *Methods Report *[30]). For all the indices we calculated scores for assessing internal consistency (Cronbach's alpha) and these indices were only used if the scores were at least 0.5. In particular, deprivation level was based on a New Zealand-specific deprivation index for small areas (NZDep2006) [34]. We considered two measures of financial stress which are correlated with each other (and the small area deprivation measure) [30], but involve significant conceptual differences [35,36]. The first question was "...because of a shortage of money, were you unable to pay any important bills on time, such as electricity, telephone or rent bills?". The second question was: "In the last six months, have you spent money on cigarettes that you knew would be better spent on household essentials like food?".

### Weighting and statistical analyses

Weighting of the results was necessary given the sampling design (eg, boosted sampling of three ethnic groups in the NZHS) and non-response for the NZHS and ITC Project survey. A full description of the weighting process is detailed in an online report [37].

Univariate analysis of all socioeconomic and smoking variables was initially conducted. We then carried out both linear and logistic regression analyses. These analyses used a conceptual framework which assumed that there would be hierarchical relationships between demographic and socio-demographic factors [38], that would dominate over smoking-related behaviours and beliefs. All models included age, gender and ethnicity, and models 2-4 included key socio-demographic variables (ie, deprivation and financial stress). For the other models we entered variables relating to smoking knowledge (of harm) and smoking behaviour (model 3); and smoking-related beliefs and attitudes (model 4). For models 3 and 4, variables were selected with a p-value of <0.05 in the univariate analyses and a forward selection procedure was used to select the final model with only statistically significant smoking-related behaviour and belief variables retained in the final models. All analyses were conducted in Stata (version 10, StataCorp, TX) and were weighted and adjusted for the complex sample design of the NZHS to make the sample representative of all New Zealand smokers.

## Results

### Smoking behaviour of these smokers

In the several months since participating in the NZHS, 12% of the sample reported that they had quit smoking. Among the continuing smokers, 95% were daily smokers, 38% smoked roll-your-own tobacco exclusively, and 23% regularly smoked a brand of "light" or "mild" factory-made cigarettes or tobacco.

### Overall support for new smokefree areas

There was a wide range of support for new smokefree areas with only 2% of respondents favouring smoking being *allowed *in all the six new settings (Figure 1). Out of a possible maximal score of 6.0 for the "Smokefree support index" (SSI) for all these settings, 59% had a score of 3.0 or higher, representing support for at least three new completely smokefree areas (ie, weighted data, Figure 1).

### Support by demographic and socio-demographic characteristics

Support for the new smokefree areas was statistically significantly higher among younger respondents and among Māori and Asian respondents, when compared to European respondents in unadjusted analyses (Table 1). The pattern for Pacific respondents was similar, but not at a statistically significant level. Support among Māori, Pacific and Asian smokers relative to European smokers remained elevated in multivariate analyses, but confidence intervals often included 1.0 (see logistic regression results below). The support was highest amongst the least and most deprived quintiles, but there were no significant differences by deprivation level. There were similar levels of strong support for smokefree areas from those with two measures of financial stress (63% and 62% respectively in the unadjusted analysis).

**Table 1:** Attitudes of respondents to new smokefree areas by demographic and socio-demographic characteristics (with all the results weighted to adjust for the complex sample design and non-response)

Variable	Strong support (row%) (Scores of 3.5 to 6.0 on the SSI)	Weak support (row%) (Scores of 0.0 to 3.0 on the SSI)	Crude odds ratios (OR) for strong support (95% CI)**
Total (n = 1376)	52.1	47.9	-

Age*			
18-24 (n = 147)	63.0	37.0	1.00 Referent
25-34 (n = 339)	55.8	44.2	0.74 (0.43 - 1.27)
35-44 (n = 353)	50.8	49.2	0.61 (0.36 - 1.03)
45-54 (n = 292)	43.7	56.3	0.46 (0.26 - 0.79)
55+ (n = 245)	48.1	51.9	0.55 (0.32 - 0.94)

Ethnicity*			
European (includes Other) (n = 620)	48.8	51.2	1.00 Referent
Māori (n = 607)	56.0	44.0	1.34 (1.00 - 1.78) (p = 0.048)
Pacific (n = 90)	59.6	40.4	1.55 (0.89 - 2.68)
Asian (n = 59)	70.1	29.9	2.46 (1.24 - 4.88)

***Small area deprivation level ***(quintiles)*			
1&2 (least deprived) (n = 121)	57.1	42.9	1.00 Referent
3&4 (n = 205)	43.9	56.1	0.59 (0.33 - 1.05)
5&6 (n = 238)	52.3	47.7	0.82 (0.46 - 1.46)
7&8 (n = 308)	50.1	49.9	0.75 (0.44 - 1.30)
9&10 (most deprived) (n = 504)	56.3	43.7	0.97 (0.58 - 1.62)

***Financial stress***			
Unable to pay any important bills on time - "yes" (n = 113), (referent = "no")	63.0	37.0	1.62 (0.93 - 2.82)
Not spending on household essentials - "yes" (n = 374) (referent = "no")	61.7	38.3	1.69 (1.22 - 2.34)

Notes:

* Based on NZHS data with the age data collected a few months prior to the ITC Project survey. For further consideration of the deprivation level and ethnicity see the Methods Section and an online *Methods Report *[30].

** All results are crude and are unadjusted for any other covariates.

### Support by knowledge of harm

Knowledge that SHS was harmful in terms of "lung cancer" and "asthma in children" was significantly associated with strong support for the new smokefree areas (Table 2). This was especially so for knowledge of "asthma in children" (OR = 2.17, 95% CI = 1.45 - 3.25). The indexes for awareness of smoking harm and for awareness of SHS harm were both in the same direction with these differences being highly significant (p < 0.001) (Table 3).

**Table 2:** Attitudes of respondents to new smokefree areas by smoking behaviours and smoking-related beliefs (all the results age-sex adjusted, weighted and adjusted for the complex design)

Variable	Strong support (column%) (Scores of 3.5 to 6.0 on the SSI) (n = 717)	Weak support (column%) (Scores of 0.0 to 3.0 on the SSI) (n = 659)	Adjusted odds ratios for strong support (95% CI)
***Quitting behaviour/addiction***			
Ever tried to quit smoking - out of those currently smoking (referent = "never tried to quit")	60.1	56.5	1.19 (0.88 - 1.63)
Perceived addiction (% at least somewhat addicted) (referent = not addicted)	80.5	87.8	0.56 (0.37 - 0.86)

***Beliefs around SHS hazards***			
Belief that smoking causes lung cancer in non-smokers from SHS (%"yes") (referent = not yes)	87.6	77.2	1.95 (1.33 - 2.84)
Belief that smoking causes asthma in children from SHS? (%"yes") (referent = not yes)	90.2	79.2	2.17 (1.45 - 3.25)

***Reasons for quitting, trying to quit ***			
"Concern about the effect of cigarette smoke on non-smokers?" ("somewhat" or "very much") (referent = "no")	70.3	52.4	2.08 (1.54 - 2.81)
"That society disapproves of smoking?" ("somewhat" or "very much") (referent = "no")	57.3	40.0	2.08 (1.56 - 2.78)
"Smoking restrictions at work?" ("somewhat" or "very much") (referent = "no")	37.9	30.1	1.45 (1.08 - 1.96)
"Smoking restrictions in public places like restaurants, cafés & pubs?" ("somewhat" or "very much") (referent = "no")	44.2	36.5	1.38 (1.03 - 1.84)
"Setting an example for children?" ("somewhat" or "very much") (referent = "no")	83.0	64.2	2.63 (1.83 - 3.79)

***Social denormalisation/smoking by friends***			
Attitude to "people who are important to you believe you should not smoke" ("strongly agree" or "agree") (referent = "not agreeing")	88.1	81.5	1.65 (1.08 - 2.52)
Zero to two friends are smokers (referent = 3+ friends who are smokers)	46.1	38.4	1.52 (1.14 - 2.04)

**Table 3:** Attitudes of respondents to new smokefree areas by indexes and scales for smoking behaviours and smoking-related beliefs

Index/scale*	Strong support [A] (crude mean score, 95% CI) (n = 825)	Weak support [B] (crude mean score, 95% CI) (n = 551)	Differences in mean scores [A - B] (Test of significance)
***Smoking/SHS knowledge of harm***			
Awareness of smoking harm (7-item index) (α = 0.69)	0.57 (0.53 - 0.61)	0.44 (0.39 - 0.49)	0.13 (p < 0.001)
Awareness of SHS harm (2-item index) (α = 0.62)	0.82 (0.77 - 0.86)	0.62 (0.56 - 0.67)	0.20 (p < 0.001)

***Smoking behaviour & smokefree rules***			
Heaviness of smoking (alternate version)	0.59 (0.36 - 0.83)	1.49 (1.24 - 1.73)	-0.90 (p < 0.001)
SHS protection (3-point scale), "How much, if at all, do you try to minimise the amount that non-smokers are exposed to your cigarette smoke?" (a high score means they try harder)	1.59 (1.52 - 1.66)	1.39 (1.30 - 1.47)	0.20 (p < 0.001)
Smokefree home (3-point scale), "Which of the following best describes smoking inside your home?" (high score is fully smokefree)	1.60 (1.53 - 1.67)	1.37 (1.30 - 1.44)	0.23 (p < 0.001)
Smokefree car (3-point scale), How you smoke "when you are in a car or other private vehicle with non-smokers" (high score is fully smokefree)	1.79 (1.73 - 1.84)	1.58 (1.51 - 1.65)	0.21 (p < 0.001)

***Smoking/SHS beliefs and attitudes***			
Smoking has affected health & quality of life (2-item index) (α = 0.68)	2.13 (2.04 - 2.21)	1.93 (1.85 - 2.01)	0.20 (p = 0.001)
Intention of quitting (4-point scale)	1.24 (1.13 - 1.34)	0.98 (0.89 - 1.07)	0.26 (p < 0.001)
Attitude to regulation (2-item index, high score is favourable toward regulation) (α = 0.51)	3.59 (3.52 - 3.66)	3.13 (3.05 - 3.21)	0.46 (p < 0.001)
Self-exempting beliefs (3-item index, high score means stronger such beliefs) (α = 0.60)	2.82 (2.74 - 2.90)	3.17 (3.09 - 3.24)	-0.35 (p < 0.001)
Overall attitude to smoking (5-point scale, high score is more positive towards smoking)	2.30 (2.22 - 2.38)	2.57 (2.49 - 2.65)	-0.27 (p < 0.001)
Smoking restrictions as reasons for quitting (2-item index covering work and restaurants/pubs, as per questions in Table 2) (α = 0.79)	1.60	1.47	0.13 (p = 0.004)

* These indices are all described in more detail in an online *Methods Report *[30].

Alpha (α) scores are for Cronbach's alpha (see *Methods*).

### Support by smoking behaviour

There were no substantive differences for many of the smoking-related behaviours we considered including by quit status and by quitting attempts (data not shown). However, those who considered that they were at least somewhat addicted to tobacco had significantly lower levels of support for the new smokefree areas (Table 2). This was also the case for the heaviness of smoking index with less support among heavier smokers (Table 3).

Those respondents who tried "a lot" to minimise exposure of others to their cigarette smoke were also more likely to be supportive (OR = 2.13, 95% CI = 1.30 - 3.49). Also, those who reported a fully effective smokefree home rule were much more supportive, as were those who "never smoke" in the car when non-smokers are present (ie, OR = 4.18, 95% CI = 1.67 - 10.46). The same pattern was found for the SHS protection index, the smokefree home index and the smokefree car index (Table 3).

### Support by smoking beliefs

Respondents indicating agreement with the following as reasons for quitting (or staying quit) were all significantly more likely to indicate strong support for new smokefree areas: concern about the effect of cigarette smoke on non-smokers; that society disapproves of smoking; because of smoking restrictions at work; because of smoking restrictions at restaurants, cafés, and pubs; and setting an example for children (Table 2). The latter was the largest association (OR = 2.63, 95% CI = 1.83 - 3.79).

Higher support was also apparent for many of the indices for beliefs shown in Table 3. These included the beliefs that smoking has affected health and quality of life; intention of quitting; having a favourable attitude toward tobacco control regulation; and reporting smoking restrictions as specific reasons for favouring quitting. In contrast, those holding stronger self-exempting beliefs around smoking and those with more positive attitudes to smoking overall, were more likely to show "weak support" for new smokefree areas.

Only one of three aspects of social denormalisation we considered showed a significant association. This was for agreement that "people who are important to you believe that you should not smoke" which was associated with strong support for new smokefree areas (Table 2). Similarly, having fewer closest friends who smoked was associated with strong support for new smokefree areas.

### Independent associations for support

The linear regression and logistic regression analyses for studying "strong support" (versus "weak support") for new smokefree areas produced very similar results. We focus here mainly on those results from the logistic regression (results in Table 4) unless otherwise stated.

**Table 4:** Logistic regression analyses on strong versus weak support for new smokefree areas

Variables*	Adjusted Odds Ratio (aOR) for strong support versus weak support (95% CI)*				
	**Model 1** **(demo-graphics)**	**Model 2** **(+ socio-demo-graphics)**	**Model 3** **(+ smoking knowledge and behaviour)**	**Model 4** **(+ smoking beliefs and attitudes)**	**Model 5** **(Model 2 plus each other variable on its own)**

***Demographic***					
Age (35-49 vs <35)	0.69 (0.49-0.97)	0.69 (0.49-0.98)	0.65 (0.44-0.95)	0.67 (0.45-1.00)	-
Age (50+ vs <35)	0.68 (0.46-0.99)	0.69 (0.47-1.01)	0.68 (0.44-1.05)	0.76 (0.48-1.20)	-
Gender (women vs men)	1.19 (0.89-1.59)	1.18 (0.88-1.57)	1.02 (0.73-1.42)	1.06 (0.75-1.51)	-
Māori vs European	1.24 (0.92-1.68)	1.19 (0.88-1.62)	1.33 (0.94-1.87)	1.22 (0.86-1.73)	-
Pacific vs European	1.45 (0.83-2.55)	1.30 (0.73-2.30)	1.43 (0.71-2.88)	1.30 (0.62-2.70)	-
Asian vs European	2.27 (1.16-4.45)	1.95 (0.97-3.92)	2.29 (0.93-5.60)	2.60 (0.99-6.83)	-

***Socio-demographic***					
Small area deprivation quintiles (increasing deprivation)	-	1.00 (0.90-1.12)	1.05 (0.93-1.19)	1.04 (0.91-1.82)	-
Financial stress: Not spending on household essentials	-	1.58 (1.13-2.21)	1.93 (1.32-2.83)	1.64 (1.11-2.43)	-

***Smoking knowledge (of harm)***					
Awareness of SHS harm (2-item index)	-	-	1.54 (1.14-2.08)	1.20 (0.87-1.65)	1.92 (1.44-2.57)

***Smoking behaviour & smokefree rules***					
Heaviness of smoking index (alternate version)	-	-	0.86 (0.79-0.93)	0.87 (0.80-0.94)	0.83 (0.77-0.89)
SHS protection scale (3-point scale), "How much, if at all, do you try to minimise the amount that non-smokers are exposed to your cigarette smoke?" (a high score means they try harder)	-	-	1.43 (1.13-1.81)	1.40 (1.09-1.78)	1.58 (1.27-1.98)
Smokefree car scale (3-point scale), How you smoke "when you are in a car or other private vehicle with non-smokers" (high score is fully smokefree)	-	-	1.70 (1.24-2.34)	1.68 (1.21-2.34)	2.24 (1.65-3.04)

***Smoking-related beliefs and attitudes***					
Reasons for quitting - concern about effect of cigarette smoke on non-smokers	-	-	-	1.24 (1.01-1.53)	1.56 (1.31-1.86)
Attitude to regulation index (2-item index, high score is favourable toward regulation)	-	-	-	1.63 (1.32-2.01)	2.04 (1.69-2.47)
Overall attitude to smoking (5-point scale, high score is more positive towards smoking)	-	-	-	0.84 (0.70-1.02)	0.70 (0.60-0.82)

Note: * The aORs in models 2 to 5 are adjusted for the demographic and key socio-demographic variables (ie, deprivation), models 3 & 4 for smoking knowledge (of harm) and behaviour variables, model 4 for smoking beliefs and attitudes, and model 5 is the same as model 2 but with each other knowledge, behaviour or belief variable included just on its own (and not adjusted for other such variables). The included variables from the univariate analyses that became not significant in the models (at p < 0.05) were subsequently omitted from the final respective models, except for those considered critical to our conceptual framework (see *Methods*).

Younger age, being female, and not being European were all associated with stronger support for new smokefree areas in multivariate models (Table 4), although 95% confidence intervals for odds ratios comparing sexes, and Pacific and Asian each with European, included 1.0.

There was significantly increased support among subjects with one form of financial stress (Table 4).

Having greater knowledge of SHS harm was associated with strong support in models 3-5, but did not reach statistical significance in the fully-adjusted model (model 4). Similarly, there were significant associations for trying hard to protect non-smokers from their cigarette smoke and for having a smokefree car (for models 3-5 eg, for model 4: aOR = 1.68, 95% CI = 1.21 - 2.34). In contrast, heaviness of smoking was associated with "weak support" (models 3-5).

Smoking beliefs and attitudes that were associated with strong support were: concern about the effect of cigarette smoke on non-smokers; having a more favourable attitude to tobacco control regulation, and that society disapproves of smoking (in the linear regression only: *β *= 0.176, p = 0.010). In contrast, having a more favourable overall attitude to smoking was associated with "weak support" (models 4 and 5). The variable around "setting an example for children" (as a reason favouring quitting) that was significant in Table 2, was not significant in the regression analysis.

## Discussion

### Main findings and interpretation

A majority of the smokers (59%) supported at least three of the six smokefree areas described, and complete lack of support for any new smokefree settings was rare (at 2%). Overall, the distribution of SSI scores was consistent with a unimodal and near normal distribution. Thus, it did not appear that the smokers responding either consistently opposed any measure, but rather that many individuals supported some, but opposed other, types of new smokefree area. For example, only 3.0% thought smoking *should be allowed *in cars with pre-school children in them, while 82.6% thought that it should be allowed in some of the outdoor seating areas of pubs (with these and other setting-specific results published elsewhere [39]).

The general picture is suggestive that the overall majority support for at least three new areas is consistent with the growing public and smoker support for smokefree laws for cars and playgrounds (with children) in a range of jurisdictions internationally (see *Background*). Of the specific hypotheses we aimed to test (see *Background*), there was general support for all three in the univariate and multivariate analyses. That is, having greater knowledge of SHS harm was independently associated with strong support for smokefree areas, as was trying hard to protect non-smokers from cigarette smoke and having concern about the effect of cigarette smoke on non-smokers. Similarly, for the second hypothesis, that having a smokefree car was associated with strong support. However, having a smokefree home was only associated with strong support in the univariate analysis. Support for tobacco control regulation was also found to be independently associated with strong support for new smokefree areas (the third hypothesis). We note that this is a similar construct to "smokefree areas laws" introducing a possible tautology, and so future work needs to explore this relationship in more detail.

While "setting an example for children" was significantly associated with strong support in the univariate analysis, this was not so in the multivariate analysis. This may reflect the fact that only a minority of smokers have young children at home, and that the impact of smoking on themselves and other adults is possibly more dominant in their thinking.

Having a form of financial stress (not spending on household essentials) was associated with strong support for smokefree areas. This might suggest that this group of deprived smokers particularly favour smokefree areas as an external means to help them cut down or to quit. This is consistent with our findings elsewhere that socio-economically deprived smokers and smokers experiencing financial stress support greater regulation of tobacco marketing, and of majority smoker support for tobacco taxation if the revenue is dedicated to smoking cessation [40].

There was a tendency towards stronger support for smokefree areas by Māori and Asian (and perhaps Pacific) smokers, compared to European smokers, including when adjusting for deprivation as shown in Table 4. This pattern may be related to various cultural reasons since it has been noted that Māori place greater priority on collective relationships [41], and so this might impact on concerns around protecting other family (whanau) members. Furthermore, there have been considerable efforts over the past decade by Māori health providers and others working in the health sector to raise awareness among Māori about SHS, including the running of mass media campaigns for a Māori audience [42], and Māori-specific resources to assist communities with implementing smokefree environments in cultural settings (eg, marae - communal meeting places [43]). Health warnings on tobacco packaging have also been in Māori language (te reo Māori) when warnings were text-only and this approach has continued with newer pictorial health warnings. There has also been an increasingly strong message from national indigenous leaders and tobacco control advocates aimed at denormalising smoking for Māori [44]. Clarifying the possible roles of these different factors could be further explored by both quantitative and qualitative studies.

Our main results may be reasonably generalisable to other developed countries which, like New Zealand, are at the tail end of the tobacco epidemic and already have in place relatively advanced smokefree environment policies. But generalisability may be much less for countries where adult smoking prevalence is high (25%+), where indoor smokefree policies are minimal or not enforced, and where there are widespread attitudes involving disrespect for the law and government authority (eg, see work by Lazuras et al [45]). Nevertheless, we note that considerable support for smokefree policies can occur in less-developed country settings [46,47].

### Strengths and weaknesses of this study

This national survey of smokers was able to explore the attitudes of smokers in a national setting where there are strong indoor smokefree laws, but where laws relating to smoking in cars and outdoor areas are largely absent. The New Zealand setting also allowed for detailed data collection by ethnicity and socio-economic position.

This study is still limited however by its cross-sectional nature (being based on wave 1 survey data). It is also likely that smokers might display some social desirability bias in their responses to surveys, and hence be more likely to articulate pro-tobacco control views. This is because smoking is becoming increasingly denormalised in many countries (eg, in New Zealand there have been reductions in socially-cued smoking following the recent expansion of indoor smokefree environment laws [23]). This bias may act both as a confounder but also as a source of (correlated and dependent) measurement error.

For example, social desirability bias is likely to vary between individuals, and within individuals cause correlated "errors" in respondent's support for new smokefree areas (dependent variable) and beliefs, attitudes and behaviour with respect to effect of SHS and smoking restrictions (independent variables). Specifically, the odds ratio of 1.40 for support for new smokefree areas by SHS protection scale in model 4 (Table 4) may be inflated, due to varying social desirability bias between individuals. In this paper, we have tried to ameliorate such bias by question wording eg, we asked about "smoking being allowed" rather than "smokefree area being required". Indeed, the nuanced and varying responses to the different questions (as discussed above), suggests that most respondents were not simply defaulting to the most pro-tobacco control answers.

Selection bias is also likely, given the non-response to the NZHS and then for those who declined to participate in the subsequent ITC Project survey. That said, in the logistic regression modelling we would presume that by adjusting for socio-demographic characteristics we are also adjusting for those variables that predict participation, and within these strata the associations of our independent and dependent variables will be more likely to represent that in the total eligible population - but this is not guaranteed. Beyond statistical imprecision, selection bias and measurement error, it is possible that we have not fully adjusted for all confounders. However, we believe this in unlikely as our ITC Project study includes good data on socio-demographics, and is restricted to smokers.

Elsewhere [39], we have described the implications of potential selection bias among survey participants, towards smokers who are more positively inclined to tobacco control measures (ie, smokers who support smokefree policies may be more likely to take part in the NZHS and then in the ITC survey). We estimated that such selection bias would have to be reasonably large to overturn key findings. "For example, there was an observed 31.9% support for smoking in playgrounds among the estimated third of all smokers first approached for interview in the NZHS that actually participated in the ITC study (i.e. third ≈ 32.6% = 67.9% [NZHS response rate] × 85.2% [NZHS consent to ITC follow up] × 56.4% [successful ITC Project survey re-contact rate]). This would have to be offset by an unobserved 58.8% support for smoking in playgrounds among the two-thirds of eligible NZHS survey smokers not included in the ITC Project study for the "true" support to be 50%. Whilst not impossible, it seems unlikely that this unobserved support might be 58.8% among non-participants compared to 31.9% among participants."

### Research and policy implications

Given the importance of smokefree areas as an evidence-based driver for improving tobacco control [1], health agencies need to continue to research public support and smoker support for new smokefree areas. A particular priority is for research around support for smokefree car laws where children are present [9], given the high level hazard from SHS in such a constrained environment. But a range of outdoor settings are probably suitable for smokefree laws, if societies wish to further minimise significant SHS exposure (eg, particularly in stadiums and semi-enclosed outdoor hospitality settings) and minimise the effect of adult modelling of smoking on youth uptake of tobacco [6].

While our cross-sectional results have limitations, the association of knowledge of the SHS hazard with smokefree area support would suggest (in line with the "health belief model" [48]) that enhancing knowledge might be a mechanism to increase smoker support for new smokefree areas. This could potentially be done by intensifying mass media campaigns that deal with SHS hazards. However, a near zero-cost alternative to these campaigns are for a government to mandate for pictorial warnings on tobacco packaging that include messages on SHS hazards to children and other adults (eg, as already used in some jurisdictions [49]). Mass media campaign campaigns that promote smokefree homes may have spill-over benefits, by enhancing public support for new smokefree laws in cars and various outdoor public places (if the association for hypothesis 2 is causal in the direction of having smokefree homes leading to support for new smokefree areas). There is evidence for such a "social diffusion model" albeit in the direction of exposure to smokefree public places being an independent predictor for implementing smokefree homes [50].

As discussed above, the association between smoker support for tobacco control regulation with strong support for new smokefree areas, is difficult to interpret. But if partly causal, an implication is that governments could further explain the need for regulatory action to their citizens and provide the rationale as to why educational measures alone are unlikely to be sufficient. If relatively higher support is shown by different ethnic groups (as in this study), then this may also encourage leadership on smokefree and other tobacco control issues, by the political leaders of such populations.

## Conclusions

For the six settings considered in this study a majority (59%) of smokers supported at least three new completely smokefree areas. Furthermore, relatively few (only 2%) of smokers favoured smoking being *allowed *in all the six new settings. Support for these new smokefree areas among Maori, Pacific and Asian smokers relative to European smokers was elevated in multivariate analyses. Identified factors associated with support included knowledge of the second-hand smoke (SHS) hazard, behaviours to reduce SHS exposure towards others, having smokefree cars and support for tobacco control regulatory measures by government. Some of these findings are consistent with the use of plausible strategies to increase support such as enhancing public knowledge of the SHS hazard (via mass media campaigns or health warnings on tobacco packaging) as means of further raising smoker support for these new smokefree areas.

## Competing interests

The authors declare that they have no competing interests.

## Authors' contributions

NW, TB, GT and RE established the NZ-arm of this study and contributed to design changes (relative to the 4-country ITC Project). DW undertook all of the statistical analyses but with input from NW, TB and RE. NW did most of the work on drafting the manuscript and HG contributed to text revisions. All authors contributed to the manuscript and approved the final manuscript.

## Pre-publication history

The pre-publication history for this paper can be accessed here:

http://www.biomedcentral.com/1471-2458/10/498/prepub
